# Supplementary material for: A Clinical Case of a Homozygous Deletion in the APOA5 Gene with Severe Hypertriglyceridemia
Source: Genes (Basel). 2022 Jun 14;13(6):1062. doi: 10.3390/genes13061062 (PMC9222921; doi:10.3390/genes13061062)
Supplement: Supplementary file 1 [file genes-13-01062-s001.zip › genes-1744522-supplementary.pdf]

Table S1. A list of the most common hereditary HTG types and associated genetic variants [21].

Condition	Prevalence	Inheritance	Genetic basis	Pathophysiology	Clinical features
Familial chylomicronemia syndrome	1 in 1 million	Autosomal recessive	Biallelic mutations in *LPL, APOC-II, APOA-V, LMF1, GPIHBP1* or *GPD1*	Defective LPL mediated clearance of chylomicrons	Serum triglycerides generally >1000 mg/dL with triglyceride to total cholesterol ratio around 10:1; recurrent pancreatitis from childhood, eruptive xanthoma, lipemia retinalis, hepatosplenomegaly
Familial hypertriglyceridemia	5-10%	No clear mendelian pattern	Polygenic with environmental influence	Increased production of triglyceride-rich VLDL particles	Serum triglycerides in 200-1000 mg/dL range with normal total cholesterol and apolipoprotein B concentrations; generally not associated with increased risk of ASCVD or pancreatitis in absence of other risk factors
Familial combined hyperlipidemia	1-2%	No clear mendelian pattern	Polygenic with environmental influence	Increased production of apolipoprotein B and associated lipoproteins	Elevated serum triglycerides, total cholesterol, or both, with elevated apolipoprotein B in patients and first degree relatives; high risk of ASCVD
Familial (type 3) dysbetalipoproteinemia	1 in 10 000	Usually autosomal recessive; rarely autosomal dominant	*APOE2/E2* genotype (AR) or rare *APOE2* mutations (AD)	Defective apolipoprotein E mediated clearance of VLDL and chylomicron remnants	Near equivalent elevations in serum total cholesterol and triglycerides (usually 300-500 mg/dL); palmar and tuberous xanthomas; secondary factors often present
Inherited lipodystrophy syndromes:					
Congenital generalized lipodystrophy	1 in 10 million	Autosomal recessive	Biallelic mutations in *AGPAT2, BSCL2, CAV1* or *PTRF*	Defective adipocyte development and differentiation leading to loss of subcutaneous fat and tendency for hepatic steatosis and VLDL overproduction	Generalized loss of body fat from birth with features of extreme insulin resistance
Familial partial lipodystrophy	1 in 1 million	Autosomal dominant; rarely autosomal recessive	Mutations in *LMNA, PPARG, PLIN1, CIDEC, LIPE, AKT2* or *ADRA2A*		Variable loss of subcutaneous fat from extremities and trunk starting in peripubertal period; features of insulin resistance

AD=autosomal dominant; ADRA2A=adrenoceptor α 2a; AGPAT2=1-acylglycerol-3-phosphate O-acyltransferase 2; AKT2=v-akt murine thymoma viral oncogene homolog 2; APOA5=apolipoprotein A5; APOC2=apolipoprotein C2; APOE2=apolipoprotein E2; AR=autosomal recessive; ASCVD=atherosclerotic cardiovascular disease; BSCL2=Berardinelli-Seip congenital lipodystrophy 2; CAV1=caveolin 1; CIDEC=cell death-inducing DFFA-like effector c; GPIHBP1=glycosylphosphatidylinositol-anchored high density lipoprotein binding protein-1; GPD1=glycerol-3-phosphate dehydrogenase 1; LIPE=hormone sensitive lipase; LMF=lipase maturation factor; LMNA=lamin A/C; LPL=lipoprotein lipase; PLIN1=perilipin; PPARG=peroxisome

proliferator-activated receptor gamma; PTRF=polymerase I and transcript release factor; VLDL=very low density lipoprotein

Table S2. List of 60 genes in our custom panel responsible for the development of primary dyslipidemias.

ABCA1	NM_005502	*APOE*	NM_000041	*HNF1A*	NM_000545	*NPC1*	NM_000271
ABCG1	NM_207174	*APOH*	NM_000042	*LCAT*	NM_000229	*NPC1L1*	NM_013389
ABCG5	NM_022436	*BSCL2*	NM_032667	*LDLR*	NM_000527	*NPC2*	NM_006432
ABCG8	NM_022437	*CAV1*	NM_001753	*LDLRAP1*	NM_015627	*PCSK9*	NM_174936
AGPAT2	NM_006412	*CAV2*	NM_001233	*LIPA*	NM_000235	*PLIN1*	NM_002666
ALMS1	NM_015120	*CAVIN1*	NM_012232	*LIPC*	NM_000236	*PLTP*	NM_006227
ANGPTL3	NM_014495	*CETP*	NM_000078	*LIPE*	NM_005357	*PPARA*	NM_005036
APOA1	NM_000039	*CH25H*	NM_003956	*LIPG*	NM_006033	*PPARG*	NM_015869
APOA2	NM_001643	*CIDEC*	NM_022094	*LMF1*	NM_022773	*PPP1R17*	NM_006658
APOA4	NM_000482	*COQ2*	NM_015697	*LMNA*	NM_170707	*PYGM*	NM_005609
APOA5	NM_052968	*CPT2*	NM_000098	*LMNB2*	NM_032737	*SAR1B*	NM_001033503
APOB	NM_000384	*CREB3L3*	NM_032607	*LPA*	NM_005577	*SCARB1*	NM_005505
APOC1	NM_001645	*GCK*	NM_000162	*LPL*	NM_000237	*SLCO1B1*	NM_006446
APOC2	NM_000483	*GPD1*	NM_005276	*MTTP*	NM_000253	*SLCO1B3*	NM_019844
APOC3	NM_000040	*GPIHBP1*	NM_178172	*MYLIP*	NM_013262	*STAP1*	NM_012108
